# Detection of K1 antigen of *Escherichia coli* rods isolated from pregnant women and neonates

**DOI:** 10.1007/s12223-014-0315-5

**Published:** 2014-04-16

**Authors:** Agnieszka Kaczmarek, Anna Budzyńska, Eugenia Gospodarek

**Affiliations:** Department of Microbiology, Ludwik Rydygier Collegium Medicum in Bydgoszcz, Nicolaus Copernicus University in Torun, 9 M. Skłodowskiej-Curie Street, 85-094 Bydgoszcz, Poland

## Abstract

The K1 antigen is an important virulence determinant of *Escherichia coli* strains and has been shown to be associated particularly with neonatal meningitis, bacteraemia and septicaemia. Thus, its detection seems to be useful, especially in the case of *E. coli* strains isolated from pregnant women and newborns. In this study, the sensitivity and specificity of the latex agglutination test (Pastorex Meningitis) for identification of *E. coli* serogroup K1 were assessed, using PCR as the gold standard. Our results showed that consistency of results between latex agglutination test and PCR amounted to 98.5 %. Therefore, Pastorex Meningitis is a good alternative to PCR and could be used for rapid K1 antigen detection, especially in local non-specialized laboratories with limited resources where PCR assay is not applied.

## Introduction

*Escherichia coli* produce more than 80 different capsular polysaccharide K antigens. One of the most extensively studied bacterial capsules is the K1 serotype of *E. coli* (Whitfield and Roberts 1999). The K1 polysaccharide is an α-2,8-linked linear homopolymer of *N*-acetylneuraminic (sialic acid; NeuNAc) and is identical to the polysaccharide antigens of group B *Neisseria meningitidis*, *Pasteurella haemolytica* A2 and *Moraxella nonliquefaciens* (Devi et al. 1991; Steenbergen et al*.* 2006; Vann et al*.* 2004). Polysialic acid is also a surface component of many fetal and adult mammalian tissues and of the neural cell adhesion molecule (N-CAM) (Bliss et al. 1996). Impossibility to present polysaccharide antigens on antigen-presenting cells (APC) and structural similarities between the K1 capsule and host tissue components may account for the poor immunogenicity of the K1 polysaccharide in humans and animals (Cisowska 2003; Vann et al*.* 2004). Molecular mimicry of the polysialic acid capsule to polysialosylglycopeptides on human fetal neuronal tissue plays an essential role in the pathogenesis of diseases caused by *E. coli* carrying the K1 capsule, particularly in infants and young children (Steenbergen et al*.* 2006; Vann et al*.* 2004). Furthermore, the K1 capsule provides the bacterium with an antiphagocytic barrier that inhibits the alternative pathway of complement (Moxon and Kroll 1990). Then, the K1 antigen has, in addition to its well-recognized serum resistance and antiphagocytic properties, a role in the traversal of *E. coli* across the blood-brain barrier as a live bacterium (Hoffman et al*.* 1999; Willis and Whitfield 2013). The K1 antigen is an important virulence determinant of *E. coli* strains and has been shown to be associated with a variety of extraintestinal diseases (Steenbergen et al*.* 2006). Capsule expression in *E. coli* has a role in virulence during urinary tract infection (UTI) and contributes to *E. coli* UTI pathogenesis by promoting biofilm-like bacterial communities in the host (Ulett et al*.* 2013). Moreover *E. coli* K1 is the most common cause of gram-negative neonatal meningitis, septicaemia, and bacteraemia (Bingen et al*.* 1996; Korczak et al. 2005; Korhonen et al. 1985; Watt et al. 2003). Despite antimicrobial therapy, these devastating diseases caused by *E. coli* K1 remain a major cause of high neonatal mortality and morbidity. Moreover, long-term neurological sequelae, including seizure disorders, hydrocephalus, physical disability, developmental delay and hearing loss, are common among more than half of the survivors (Korczak et al*.* 2005). It is therefore important to identify the K1-encapsulated *E. coli* rapidly and reliably.

Only a few studies (Cross et al*.* 1984; Devine et al*.* 1990; Gross et al*.* 1977) have examined the use of different methods for the detection of the K1 capsular polysaccharide among clinical isolates of *E. coli*, and in the available articles, there is a lack of research results of the K1 antigen detection using Pastorex Meningitis. Therefore, for the purpose of obtaining more information about the reliable detection of the K1 antigen, we analysed the effectiveness of K1 antigen identification using the latex agglutination test—Pastorex Meningitis—compared to PCR.

## Materials and methods

### Bacterial strains

A total of 134 genetically unrelated *E. coli* isolates were analysed in this study. Genotypic relatedness of these strains was assessed using pulsed-field gel electrophoresis (PFGE) analysis (data not shown). One hundred and seven *E. coli* strains were obtained from pregnant women from three different sources: faeces (*n* = 74), vagina (*n* = 21) and urine (*n* = 12) samples. The study also included 27 *E. coli* strains isolated from newborns: 19 were isolated from the nasal cavity, 5 were isolated from the urine and 3 were isolated from the faecal samples. All strains were isolated from June to September 2008, from patients hospitalized at Dr. J. Biziel University Hospital No. 2, L. Rydygier Collegium Medicum in Bydgoszcz at Nicolaus Copernicus University in Torun, Poland. Permission to carry out the research was given by the Bioethics Committee of Collegium Medicum in Bydgoszcz at Nicolaus Copernicus University in Torun. Bacteria were cultured on MacConkey agar and identified by a biochemical method (ID 32E, bioMérieux).

### K1 antigen detection

Two methods were used for K1 antigen determination: latex agglutination test (Pastorex Meningitis, Bio-Rad) and PCR-based detection of *neuC* (K1-specific gene).

### Latex agglutination method

Latex agglutination test for the K1 antigen was performed using latex particles coated with mouse monoclonal antibodies specific for *N. meningitidis*/*E. coli* K1 according to the manufacturer’s instruction. In the presence of the K1 antigen, the latex particles agglutinated. In the absence of this antigen, latex particles remained in a homogeneous suspension. Latex containing the polysaccharide antigens of *N. meningitidis* A, C, B and Y/W135; *Haemophilus influenzae* b; *Streptococcus* B and *Streptococcus pneumoniae* controlled the immunoreactivity of latex sensitized with the mouse monoclonal antibody specific for *N. meningitidis* group B/*E. coli* K1, whereas latex sensitized with IgG immunoglobulins from a non-immunized rabbit controlled the absence of unspecific agglutination.

### Isolation of the bacterial DNA and PCR amplification

DNA was extracted from bacteria using a Genomic Mini Purification kit (A&A Biotechnology) according to the manufacturer’s instructions. The DNA extract was stored at −20 °C prior to PCR. The PCR assay was performed using primers specific for the gene encoding K1 antigen (*neuC*) (neuC1 5′-AGG TGA AAA GCC TGG TAG TGT G-3′, neuC2 5′-GGT GGT ACA TCC CGG GAT GTC-3′) (Moulin-Schouleur et al*.* 2007). The detected virulence gene was amplified in a total volume of 25 μL containing 1.5× PCR buffer, 2 mM MgCl_2_, 5 nmol each dNTP (Promega), 12.5 pmol each primer (Laboratory of DNA Sequencing and Oligonucleotide Synthesis, IBB Polish Academy of Sciences, Poland), 1 U Taq DNA polymerase (Promega) and 50 ng of DNA. The amplification was done in the GeneAmp PCR System 2700 thermal cycler (Applied Biosystems). The PCR steps were as follows: initial denaturation at 94 °C for 3 min, followed by 30 cycles of denaturation at 94 °C for 1 min, annealing at 58 °C for 1 min and extension at 72 °C for 1 min, followed by a final 10-min extension at 72 °C. The reaction conditions were adapted from the method of Moulin-Schouleur et al. (2007). After amplification, the PCR products were separated by electrophoresis in a 1.5 % agarose gel, stained in ethidium bromide solution and visualized with a GelDoc 2000 gel documentation system (Bio-Rad). During each run, the DNA template from the *E. coli* BEN2908 strain and Molecular Grade Water (Sigma) instead of DNA were included as positive and negative controls, respectively (Germon et al. 2005). The *E. coli* BEN2908 strain was provided by Dr. Pierre Germon (Unité Infectiologie Animale et Santé Publique, France).

## Results

**Fig. 1:**
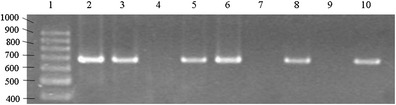
Polymerase chain reaction products for the *neuC* (676 bp) gene. Lines: *1*, DNA marker (GeneRuler^TM^100 bp DNA Ladder); *2*–*8*, tested DNA samples; *9*, negative control; *10*, positive control for the *neu*C (676 bp) (*E. coli* strain BEN2908) gene

Latex agglutination test (Pastorex Meningitis) and PCR were used to identify the *E. coli* K1 strains. Confirmation of the results of the K1 surface antigen identification in *E. coli* strains using latex agglutination test and PCR was obtained for 132 (98.5 %) of the 134 tested strains. The latex agglutination method falsely identified two strains—one as K1 negative (sensitivity 98.5 %) and one as K1 positive (specificity 98.5 %)—in comparison with the PCR results (Table 1). Examples of the PCR amplification results for the gene analysed in this study are shown in Fig. 1.

**Table 1:** Discrepancies between the Pastorex Meningitis test and PCR assay in identification of *E. coli* K1 strains

No. of isolates	Results	
	Pastorex Meningitis	PCR
66	K1+	K1+
66	K1−	K1−
1	K1+	K1−
1	K1−	K1+

*K1+* presence of K1 antigen/*neuC* gene, *K1−* lack of K1 antigen/*neuC* gene

## Discussion

*E. coli* is the principal gram-negative rod responsible for meningitis and sepsis in newborn and premature infants. Most of the *E. coli* strains isolated from the cerebrospinal fluid or the blood of patients with these infections possess the K1 capsular polysaccharide antigen as an essential virulence determinant. It was shown that 78–92 % of neonatal meningitis *E. coli* (NMEC) strains were K1 positive (Bingen et al. 1997; Johnson et al*.* 2002; Korhonen et al*.* 1985; Obata-Yasuoka et al*.* 2002; Watt et al*.* 2003). The mean prevalence of the K1 antigen among the NMEC strains was higher than that among the neonatal septicaemia strains (59–70 %) (Bingen et al. 1996; Obata-Yasuoka et al. 2002; Watt et al. 2003).

The *E. coli* strains involved in a large number of neonatal diseases are thought to originate from the natural microflora of pregnant women. Vaginal colonization with *E. coli* seems to be an important step in neonatal infection, and it was observed in 24–31 % of pregnant women. About half of all vaginal *E. coli* strains express the K1 antigen (Obata-Yasuoka et al*.* 2002; Watt et al*.* 2003). Also, *E. coli* strains isolated from the intestinal microflora of asymptomatic pregnant women often possess the capsular K1 antigen (Watt et al*.* 2003). The transmission of *E. coli* strains from the mother to the newborn frequently occurs during passage of the neonate through the vaginal canal. Thus, programmes to screen for vaginal and rectal colonization of pregnant women by *E. coli*, particularly those with the K1 antigen, may be effective for infection prevention.

The K1 antigen may be identified by immunological techniques such as seroagglutination, antiserum-agar diffusion test or immunoelectrophoresis. These methods identify the K1 capsule by the use of an antiserum to *N. meningitidis* group B/*E. coli* K1 polysaccharide (Bingen et al*.* 1997; Cisowska et al*.* 2001; Cross et al*.* 1984; Obata-Yasuoka et al*.* 2002; Siitonen et al*.* 1993). Due to the fact that the K1 antigen is a poor immunogen, its detection by serological means is difficult and largely confined to specialist laboratories. An easier technique for detecting this antigen depends on the use of K1-specific bacteriophages (Cisowska et al*.* 2001; Devine et al*.* 1990; Gross et al*.* 1977; Obata-Yasuoka et al*.* 2002; Siitonen et al*.* 1993). It is also possible to use the latex agglutination test and PCR amplification in order to identify the *E. coli* K1 strains (Kaczmarek et al*.* 2012; Moulin-Schouleur et al*.* 2007; Obata-Yasuoka et al*.* 2002; Siitonen et al*.* 1993; Watt et al*.* 2003). Previous studies compared the use of bacteriophage sensitivity, seroagglutination, immunodiffusion and immunoelectrophoresis techniques for the detection of the K1 capsular polysaccharide among isolates of *E. coli* (Cross et al*.* 1984; Devine et al*.* 1990; Gross et al*.* 1977)*.* The results of these studies indicate that there is no one method for K1 detection which can be considered to be the standard.

In the present study, for the purpose of obtaining more information about a reliable method for detection of the K1 antigen, we analysed the effectiveness of the K1 antigen identification using latex particles coated with mouse monoclonal antibodies specific for *N. meningitidis* group B/*E. coli* K1 (Pastorex Meningitis, Bio-Rad) compared to PCR. We conclude that Pastorex Meningitis is almost as accurate and reliable as PCR. Moreover, it is a rapid test that can be completed in 15 min as compared to the 2–3 h required for PCR assay. The Pastorex latex agglutination test is very easy to interpret, similar to PCR assay. In the presence of the K1 antigen, latex particles agglutinate for up to 2 min. In the absence of this antigen, they remain in a homogeneous suspension, without visible aggregates. There was good agreement among the results obtained with this latex agglutination test and the PCR assay. The sensitivity and specificity of Pastorex Meningitis amounted to 98.5 %. The latex agglutination method falsely identified two strains. A false-negative result may be caused by lack of expression of the gene encoding the K1 antigen. However, the latex agglutination test is reproducible and less labour-intensive than other methods used for identification of the *E. coli* K1 antigen.

In conclusion, Pastorex Meningitis is a good alternative to PCR, especially in local, non-specialized laboratories with limited resources where PCR assay is not applied. Moreover, Pastorex can be performed with rapid results, which are necessary to start the appropriate treatment as soon as possible. It is important to decrease the mortality rate from invasive infection with the K1-encapsulated *E. coli*, especially among neonates.
